# Anatomical Models

**Published:** 1831

**Authors:** 


### LXIII.

### ANATOMICAL MODELS.

WE have again to invite the attention of our brethren to the anatomical models imported into this country from Germany, by Mr. SCHLOSS, who has removed from Southampton Buildings, to a more central, if not more fashionable situation, No. 103, St. Martin's Lane, Charing Cross. Mr. S. has been encouraged to undertake the importation of such novelties in foreign literature, arts, and sciences, as are deemed worthy of the approbation of the profession in this country. We have no doubt that he will receive the patronage of the public in these laudable attempts to accommodate them.

